# Supplementary material for: Combination of Multiple Microsatellite Analysis and Genome-Wide SNP Genotyping Helps to Solve Wildlife Crime: A Case Study of Poaching of a Caucasian tur (Capra caucasica) in Russian Mountain National Park
Source: Animals (Basel). 2021 Nov 30;11(12):3416. doi: 10.3390/ani11123416 (PMC8697997; doi:10.3390/ani11123416)
Supplement: Supplementary file 1 [file animals-11-03416-s001.zip › Table S1.pdf]

Table S1. The concentrations of double-stranded DNA and the absorption ratio of OD260/OD280 for studied items.

**No**	**Sample ID**	**Absorption ratio of OD260/OD280**	**Concentrations of dsDNA, ng/ µL**
1	Sample No. 1	1.92	149.6
2	Sample No. 2	1.92	164.0
3	Sample No. 3	1.87	137.6
4	Sample No. 4	1.91	82.8
5	Sample No. 5	1.83	444.0
6	Sample No. 6	1.46	5.6
7	Sample No. 7	1.35	0.1
