# Supplementary figures and images for: Combination of Multiple Microsatellite Analysis and Genome-Wide SNP Genotyping Helps to Solve Wildlife Crime: A Case Study of Poaching of a Caucasian tur (Capra caucasica) in Russian Mountain National Park
Source: Animals (Basel). 2021 Nov 30;11(12):3416. doi: 10.3390/ani11123416 (PMC8697997; doi:10.3390/ani11123416)

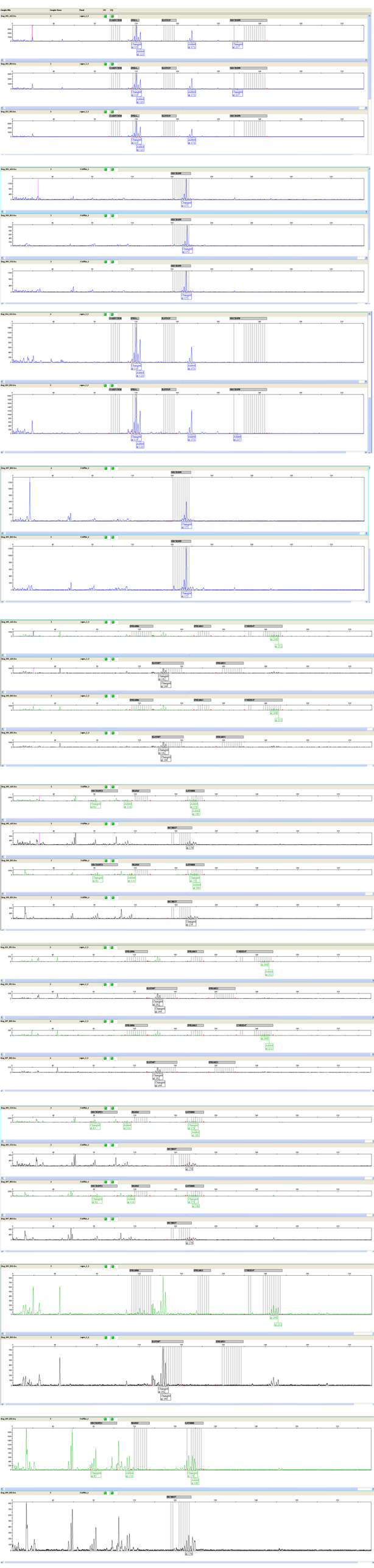

Supplement: Supplementary file 1 [file animals-11-03416-s001.zip › Fig_S1.pdf]
